# CRISPR/Cas- and Topical RNAi-Based Technologies for Crop Management and Improvement: Reviewing the Risk Assessment and Challenges Towards a More Sustainable Agriculture

**DOI:** 10.3389/fbioe.2022.913728

**Published:** 2022-06-28

**Authors:** Fabiano Touzdjian Pinheiro Kohlrausch Távora, Francisco de Assis dos Santos Diniz, Camila de Moraes Rêgo-Machado, Natália Chagas Freitas, Fabrício Barbosa Monteiro Arraes, Eduardo Chumbinho de Andrade, Leila Lourenço Furtado, Karen Ofuji Osiro, Natália Lima de Sousa, Thiago Bérgamo Cardoso, Liliane Márcia Mertz Henning, Patrícia Abrão de Oliveira Molinari, Sérgio Enrique Feingold, Wayne B. Hunter, Maria Fátima Grossi de Sá, Adilson Kenji Kobayashi, Alexandre Lima Nepomuceno, Thaís Ribeiro Santiago, Hugo Bruno Correa Molinari

**Affiliations:** ^1^ Department of Phytopathology, University of Brasília, Brasília, Brazil; ^2^ Embrapa Agroenergy, Brasília, Brazil; ^3^ Embrapa Genetic Resources and Biotechnology, Brasília, Brazil; ^4^ Embrapa Cassava and Fruits, Cruz Das Almas, Brazil; ^5^ IPADS-Balcarce (UEDD INTA-CONICET), Balcarce, Argentina; ^6^ SEMPRE AgTech, Chapecó, Brazil; ^7^ Embrapa Soybean, Londrina, Brazil; ^8^ USDA-ARS, U.S. Horticultural Research Laboratory, Fort Pierce, FL, United States

**Keywords:** exogenous dsRNA, genome editing, gene silencing, nanotechnology, offtargets, public acceptance, regulatory aspects, toxicity

## Abstract

Clustered regularly interspaced short palindromic repeats (CRISPR)/CRISPR-associated gene (Cas) system and RNA interference (RNAi)-based non-transgenic approaches are powerful technologies capable of revolutionizing plant research and breeding. In recent years, the use of these modern technologies has been explored in various sectors of agriculture, introducing or improving important agronomic traits in plant crops, such as increased yield, nutritional quality, abiotic- and, mostly, biotic-stress resistance. However, the limitations of each technique, public perception, and regulatory aspects are hindering its wide adoption for the development of new crop varieties or products. In an attempt to reverse these mishaps, scientists have been researching alternatives to increase the specificity, uptake, and stability of the CRISPR and RNAi system components in the target organism, as well as to reduce the chance of toxicity in nontarget organisms to minimize environmental risk, health problems, and regulatory issues. In this review, we discuss several aspects related to risk assessment, toxicity, and advances in the use of CRISPR/Cas and topical RNAi-based technologies in crop management and breeding. The present study also highlights the advantages and possible drawbacks of each technology, provides a brief overview of how to circumvent the off-target occurrence, the strategies to increase on-target specificity, the harm/benefits of association with nanotechnology, the public perception of the available techniques, worldwide regulatory frameworks regarding topical RNAi and CRISPR technologies, and, lastly, presents successful case studies of biotechnological solutions derived from both technologies, raising potential challenges to reach the market and being social and environmentally safe.

## 1 An Overview of Plant Breeding: From Ancient Times to Genetic Manipulation Associated With Molecular Breeding

The use of improved genotypes in agriculture started 10,000 years ago with the process of crop domestication when humans began to adapt wild plant species for cultivation as food plants (Doebley et al., 2006). For many years, conventional plant breeding has been performed by artificial crossing or induced random mutagenesis, and the selection of parents and descendants is based majorly on the phenotype, hence in the absence of molecular and physiological basis of enhanced traits (Jorasch, 2019). These breeding approaches, although time-consuming, labor-intensive, and randomly oriented to some extent, continue to deliver crop varieties supporting demands for increased agricultural production (Scheben et al., 2017).

In the last 30 years, biotechnology tools have allowed the development of desirable genotypes in less time and generally at a lower cost compared to conventional breeding. Modern agriculture has profited from advances in molecular biology and next-generation sequencing (NGS) technologies for high throughput sequencing, which revolutionized genetic plant breeding, with emphasis on transgenic technology, molecular markers, and genomic selection (Kim et al., 2020; Thudi et al., 2021).

Transgenic breeding has been the most frequent technique applied for plant genetic manipulation in history, allowing desirable target genes to be introduced into the plant genome ideally without making other unintended genetic changes (Qaim, 2020). These early developments showed the capability of genetically engineering a plant genome and inspired other breeding approaches such as gene silencing. The first report of gene silencing in plants was demonstrated in 1989 in tobacco plants (Matzke et al., 1989), and a subsequent study showed that the integration of transgenes homologous to plant endogenous genes could result in suppression of both expressed genes, a process called co-suppression (Napoli et al., 1990). Later, Fire et al. (Fire et al., 1998) used the nematode *Caenorhabditis elegans* to show for the first time that the suppression of target transcripts expression is triggered by double-stranded RNA (dsRNA) molecules, a mechanism known as “RNA interference” (RNAi). Since then, several components of the RNAi pathway were identified, and the practical use of RNAi-based GMOs has advanced rapidly (Saurabh et al., 2014). However, unlike GMO plants that are generally modified to express a specific protein, RNAi-based GMO plants have been modified to express dsRNA molecules that enable specific silencing of target genes on the plant or pathogen/pest genomes (Arpaia et al., 2020), a strategy termed host-induced gene silencing (HIGS). According to Ghag et al. (Ghag, 2017), HIGS was an innovative concept of RNAi technology for effective silencing of one or a few genes with agronomic importance. This technology has many potential applications in agriculture, including enhancing resistance against biotic and abiotic stresses, improving industrial and nutritional quality, delayed ripening, male sterility, plant architecture modification, and removal of allergens and toxins (Rajam, 2020).

RNAi pathways are natural mechanisms present in almost all eukaryotic organisms. Basically, these pathways work through processing long dsRNA into called small interfering RNA (siRNA) or micro-RNA (miRNA) molecules, which are responsible for recognizing the target messenger RNA (mRNA), as well as guiding the DNA and histone modifications or chromatin remodeling, leading to target gene silencing (Wilson and Doudna, 2013). RNAi-based GMOs have become key elements for plant breeding, due to their ability to modulate gene expression in a sequence-specific manner. However, there are great constraints and delicate issues related to the use of transgenics—including the transgenic approach of RNAi-based technology—that have negatively impacted the development of new GMO crops, such as high costs, negative perception of some consumers, long timelines to succeed, restrictive regulatory framework, and the lack of genetic transformation protocols for many crop species (Scheben et al., 2017; Fletcher et al., 2020). Despite the mentioned rapidness of the transgenic approach, the approval of a new GM plant takes, on average, 10–12 years of successive biochemical, molecular, environmental, and animal health-related trials, according to the regulation adopted by each country (Qaim, 2020).

In this context, since the early 2000s, the use of RNAi-based non-transgenic approaches (e.g., exogenous and self-deliverable dsRNA molecules) has been explored in agriculture, mostly for plant protection against pathogens and pests (Tenllado et al., 2003; Rego-Machado et al., 2020; Kalyandurg et al., 2021). This strategy, currently known as spray-induced gene silencing (SIGS), has been attempted as a potential and alternative biotechnological tool for transgenic plants, due to its appealing features, being too much faster, cheaper, easier to handle, and capable to encompass a broader range of target organisms (Rank and Koch, 2021), while avoiding plant transformation/screening steps, and biosafety issues in some extent. Furthermore, this approach holds enormous potential to meet the increasing public demand for reducing agrochemical applications toward more sustainable and agroecological production. In addition, SIGS has been shown to be more efficient under lab conditions compared to the HIGS strategy (Koch et al., 2016). Nowadays, there is mounting evidence suggesting that topically-applied dsRNAs molecules are effective in silencing target genes aiming at plant resistance against a broad range of biotic factors (Dubrovina and Kiselev, 2019; Dalakouras et al., 2020; Das and Sherif, 2020).

Additionally, advances in genomics studies with nuclease enzymes have allowed the emergence of equally revolutionary novel non-transgenic tools that are used in site-directed genome editing for precision plant breeding, also not necessarily involving the integration of exogenous sequences into the plant genome. Based on the mode of action of these gene-editing tools, DNA is modified, inserted, replaced, or deleted in the plant genome at specific locations using sequence-specific nucleases, leading to gene modification at target sites. These genomic editing tools can be used to improve multiple traits simultaneously, controlled by multiple loci of the genome (Rodríguez-Leal et al., 2017), facilitating the development of commercial products, which is often difficult using conventional genetic breeding techniques.

In this decade, the most widely used gene-editing technology is the clustered regularly interspaced short palindromic repeats (CRISPR)/CRISPR-associated gene (Cas) system, an adaptive immunity mechanism found in bacteria and archaea against bacteriophages and mobile genetic elements, which was transformed as a genome editing biotechnological tool in 2012 (Jinek et al., 2012). This tool relies on a special site of the bacterial genome called CRISPR locus, which is a gene array composed of spacers acquired from the invader’s exogenous DNA and integrated between small bacterial palindromic repeats. Flanking the CRISPR locus there are genes encoding Cas nucleases, responsible for cleavage of exogenous DNA upon new infection by the same invader. The spacers are transcribed into small guide RNAs that once complexed with Cas nucleases direct the breakdown of the intruder DNA (Marraffini, 2015). The use of CRISPR/Cas in plant breeding allows the segregation of system components (e.g., Cas protein and guide RNA—gRNA) out of the host genome, post-target gene editing, enabling the generation of non-transgenic crops. Moreover, for this purpose, the system has been experimentally optimized, and a transgene-free approach to the technology can be performed which usually involves the use of a ribonucleoprotein (RNP) complex made only by the gRNA and Cas nuclease protein transcribed *in vitro* (Zhang et al., 2021a).

**FIGURE 1:**
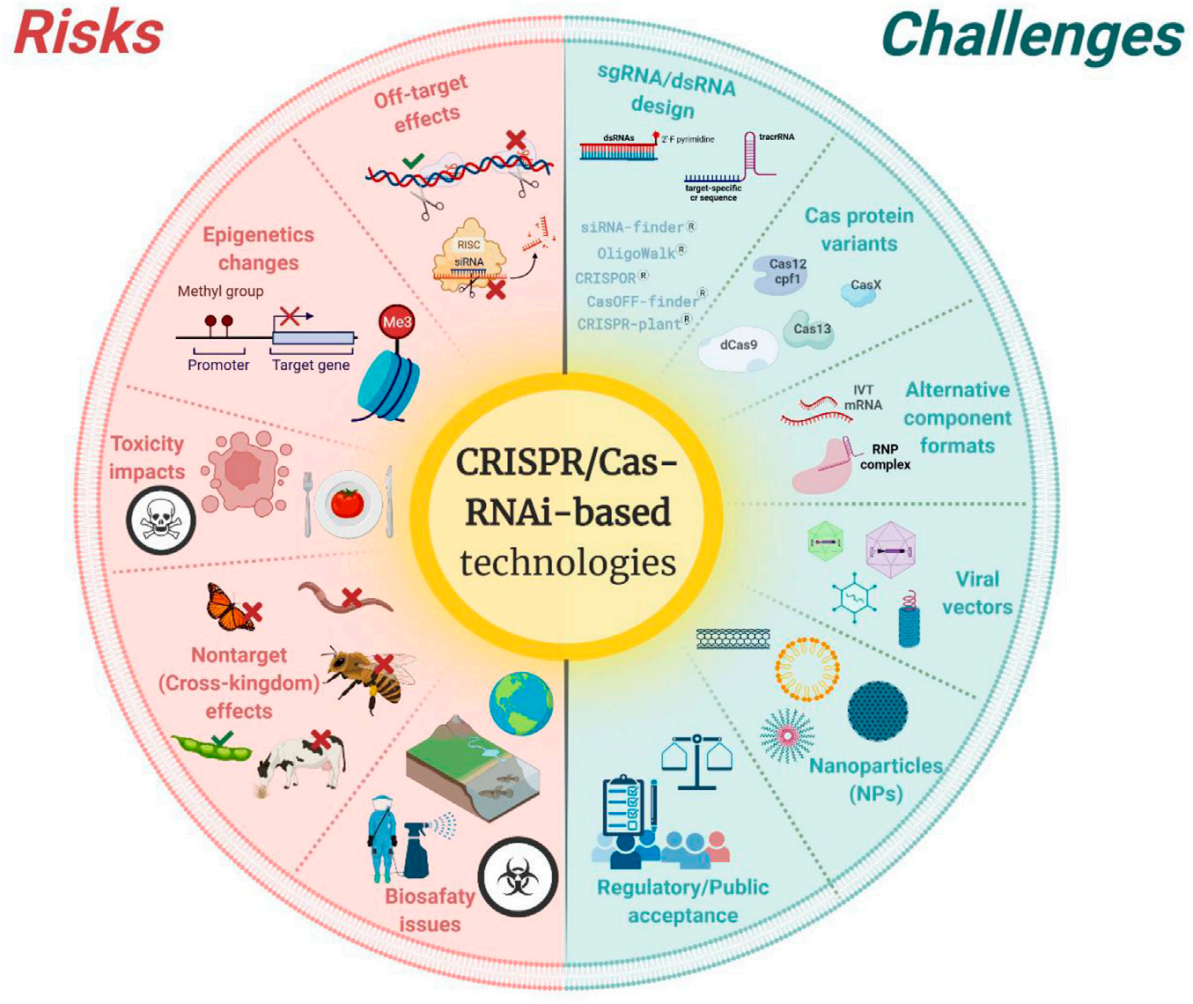
Summary of the main risks (on the left—in red color) and challenges (on the right—in green color) addressed in the text and related to the application of Clustered regularly interspaced short palindromic repeats (CRISPR)/CRISPR-associated gene (Cas)- and RNA interference (RNAi)-based technologies in agriculture. Created with Biorender software at https://biorender.com.

Indeed, both technologies—RNAi and CRISPR/Cas—have the power to revolutionize plant research and breeding (Younis et al., 2014; Guo et al., 2016; Ricroch and Hénard-Damave, 2016). In this review, we present an up-to-date panorama on advancements and breakthroughs of both technologies for breeding and plant protection, as well as provide a broad perspective on the risks, challenges, public perception, and regulatory aspects concerning the applications of non-transgenic approaches of both genetic engineering technologies in modern agriculture. In Figure 1, we summarized the main risks and challenges related to both technologies, which will be discussed further in this review.

## 2 Genome Editing Technology Focuses on CRISPR/Cas Technology

### 2.1 CRISPR/Cas in Agriculture

CRISPR/Cas has been used in different crops since 2013, introducing into them agricultural traits of great value, such as yield, quality, and biotic-/abiotic-stress resistance (Shan et al., 2013; Zhang et al., 2020). This technology holds an enormous potential to address numerous concerns involving cost, time, and complex biosafety issues, typical characteristics of the transgenic strategy. Furthermore, the ever-expanding CRISPR/Cas toolbox has allowed a myriad of applications in plants, including the knockout and knock-in of target genes, modulation (i.e., inhibition or activation) of gene expression, genome base editing, among others (Zhu et al., 2020).

Differently from other genome editing-based technologies, such as zinc-finger nuclease (ZFN) and transcription activator-like effector nuclease (TALEN), the use of CRISPR/Cas does not depend on engineered proteins, and it is essentially based on RNA/DNA hybrids, in which its target specificity relies on a short stretch of RNA, providing higher versatility, lower costs and an easier building process. Furthermore, this technology enables the editing of multiple genome sites simultaneously (Xiong et al., 2015), and also introduces mutations directly into elite crop varieties, bypassing limitations like narrowed natural genetic variability, and time-consuming processes of backcrossing to reconstruct the elite genetic background as in conventional breeding technique (Scheben et al., 2017; Rato et al., 2021), being especially useful for crops with rare resistance sources, long life cycles, and polyploid genomes.

The gene regulation can be modulated by the use of catalytically inactive Cas9 variants (e.g., dead Cas9—dCas9) or orthologs. These enzymes are capable of binding to specific DNA sequences mediated by gRNA without causing double-strand breaks on the DNA molecule. (Lowder et al., 2018; Papikian et al., 2019). The dCas9 fused to transcription regulatory domains, such as VP64 or SRDX, or epigenetic modulators can be used for activation or repression through CRISPR interference (CRISPRa or CRISPRi, respectively), expanding its range of applications (Moradpour and Abdulah, 2020). For example, dCas9-VP64 and dCas9-TV systems increased the expression of the UDP-glucose flavonoid glycosyl-transferases (UFGT) gene in grape cells (Ren et al., 2022). dCas9 can also promote/inhibit enhancers in promoter regions of genes due to the interference in chromatin structure, consequently modulating the gene expression (Morgan et al., 2017). Another way of modifying the gene expression is the cleavage and degradation of RNA-targeting using Cas13a and Cas13b. Editing RNA with CRISPR/Cas13 is a novel and emergent tool in plants and is currently being used mainly for developing resistance to viral diseases in plants (Zetsche et al., 2015).

Beyond the coding (CDS) and promoter regions, other regulatory elements are also good targets for genome editing aiming to modulate gene expression, such as polyadenylation signals, alternative transcription initiation sites, and upstream open reading frames (uORFs). Usually responsible for reducing the translation, uORFs are situated in the 5′ untranslated regions (UTRs) of mRNAs, and when edited can promote the upregulation of gene expression. For example, the CRISPR knockout of gene’s uORF region resulted in an increase in gene translation in lettuce (*Lactuca sativa*) and strawberry (*Fragaria vesca*) plant crops, leading to a high content of ascorbate and sweetness in the edited plants, respectively (Zhang et al., 2018; Xing et al., 2020).

### 2.2 Risks and Challenges Involving CRISPR/Cas Technology

#### 2.2.1 Unintended Off-Target Effects (General Immune Response)

During the activity of CRISPR/Cas machinery, the gRNA can direct the Cas protein to other regions and consequently lead to unintentional cleavage of DNA sequence, a process known as off-target effect. Shahriar et al. (Shahriar et al., 2021) classified the off-targets into two types: 1) sequences sharing high similarities to the target, and 2) irrelevant genomic off-target sites. These off-target mutations are of great concern mainly in the clinical-therapeutic area (Zhang et al., 2015), which restricts its application due to technical and ethical issues. In major crop plants, several studies have reported the incidence of unwanted changes in the genome, but at low rates (<10%) (Peng et al., 2017; Tang et al., 2018; Young et al., 2019; Graham et al., 2020; Jin et al., 2021), suggesting a remarkable specificity of CRISPR/Cas system in the plant genome, or either a flaw in the currently available off-target detection methods (Bortesi and Fischer, 2015; Hajiahmadi et al., 2019). Nevertheless, when an off-target effect is detected, it is generally located at genomic spots exhibiting great similarity to the target sites (Lawrenson et al., 2015; Tang et al., 2018). Some *in vitro* and *in vivo* methods have been developed to detect these mutations, such as Digenome-seq (Kim D. et al., 2015), GUIDE-seq (Tsai et al., 2014), SITE-seq (Cameron et al., 2017), CIRCLE-seq (Tsai et al., 2017), and DISCOVER-seq (Wienert et al., 2019). A gold standard recommendation would be performing genome-wide NGS for the identification of these potential off-target mutations, however, it seems not to be practical/feasible in most cases (Hahn and Nekrasov, 2019; Shillito et al., 2021), especially for polyploid crops. Consequently, an underestimation of off-target mutation rates might be occurring, although not likely.

#### 2.2.2 Epigenetic Consequences

Epigenetic phenomena consist of a complex gene expression regulation process for the maintenance of a precise state of gene activation/repression in a given cell (Urnov and Wolffe, 2001). Such a sophisticated and fine-tuned mechanism involves a series of alterations in DNA molecules, including chemical modification of DNA structure (e.g., methylation), modification in histone proteins (closely associated with the gene locus), and chromatin remodeling, without altering DNA primary sequence (Jaenisch and Bird, 2003). Although epigenetic characteristics can influence cleavage by facilitating or hindering DNA accessibility, unintended effects on the genome beyond off-target mutations caused by the use of CRISPR technology are still poorly explored. Lee et al. (2020) analyzed the DNA methylation profiles in promoters of naturally hyper and hypomethylated genes from *Arabidopsis thaliana* that underwent genome editing through CRISPR/Cas. Edited and wild-type plants showed the same epigenetic profile by sequencing the next generation of bisulfite-converted DNA, concluding that CRISPR genome editing did not result in unintended epigenetic changes. However, only one work was carried out in the area and one epigenetic mechanism was evaluated. DNA methylation is the most common epigenetic marker in plants and occurs mainly by the insertion of a methyl group (CH_3_) on the fifth carbon of cytosines in CpG (cytosine-phosphate-guanine) dinucleotides (Laird, 2010; Yong et al., 2016). Meantime, epigenetic information is also mediated by post-translational histone modifications (MPTHs) and processing mechanisms of non-coding RNAs (ncRNAs) (Bossdorf et al., 2010).

Another important point to be raised is the DNA accessibility in target regions by CRISPR technology. Studies have shown that the level of accessibility to the loci through DNA methylation or chromatin structure can influence the efficiency of on-target gene editing (Jensen et al., 2017; Verkuijl and Rots, 2019; Strohkendl et al., 2021). The chromosome with high compaction can lead to low DNA accessibility to non-specific Cas9 interactions (Chitra et al., 2019). Nucleosomes are known to inhibit PAM (protospacer adjacent motif) site recognition, reducing the rates of Cas nuclease cleavage *in vitro* (Verkuijl and Rots, 2019; Strohkendl et al., 2021). Additionally, there is a positive correlation between chromatin opening and the efficiency of mutagenesis by the CRISPR system (Uusi-Mäkelä et al., 2018). For example, a transcriptional activation domain fused to Cas9 improved the genome editing efficiency in condensed and relaxed chromatin regions in rice (Liu et al., 2019). Given the above, in addition to PAM recognition and complementarity between gRNA and target DNA, DNA accessibility should also be considered an important factor for genome editing efficiency.

#### 2.2.3 Toxicity Impacts on Human/Animal Health

The toxicity associated with CRISPR/Cas application may be caused by its components, the exposure period, and/or depending on the delivery methods. Several studies involving different organisms, such as prokaryotes (Jiang et al., 2014, 2017; Cho et al., 2018; Markus et al., 2019) and pluricellular eukaryotes (Ihry et al., 2018; Li et al., 2018; Rosenblum et al., 2020), have shown that either induced double-strand break (DSB) and heterologous Cas9 protein expression can impair cell growth that leads to an abnormality in cell morphology and/or trigger cell death. To date, no reports of Cas9-associated toxicity have been found in plants (Dey, 2021). Since the first applications of CRISPR technology in plant cells, researchers have shown that whole plants can be regenerated by tissue culture from edited cells, suggesting that the CRISPR system components are not toxic to plants (Hahn and Nekrasov, 2019). However, depending on the adopted CRISPR/Cas strategy and the target chosen, serious pleiotropic effects may occur (Zhu et al., 2020). For example, the knockout of plant susceptibility (-*S*) genes, associated with pathogen compatibility, but also engaged with multiple crucial pathways, often lead to plant fitness penalties, including physiological and growth tradeoffs (Kale et al., 2019; van Butselaar and Van den Ackerveken, 2020; Kieu et al., 2021). In addition, studies have evaluated the toxicity of CRISPR/Cas components not only in plants but also in humans. Regarding the exposure of Cas protein in humans, an interesting study conducted by El-Mounadi et al. (El-Mounadi et al., 2020) concluded that exposure of human beings to Cas9 proteins took place long before the emergence of genomic editing tools. In the comparative genomic analyses, the authors detected more than 80% similarity between *Streptococcus pyogenes* (*Sp*Cas9) amino acid sequence with commensal/pathogenic bacteria such as *Streptococcus dysgalactiae* subsp. *equisimilis*, *Staphylococcus aureus*, *Klebsiella pneumonia*, and *Streptococcus canis*, are commonly found in the environment or even in foods intended for human consumption. Furthermore, *Sp*Cas9 has homologs in Gram-positive and Gram-negative bacteria naturally found in different niches throughout the human body (Louwen et al., 2014). Hence, edited plants containing Cas9 integrated into the genome probably do not represent a potential risk to human health.

Nevertheless, the mode of delivery of CRISPR/Cas system components seems to stand out as one of the main factors of toxicity in plants. For example, nanoparticle (NP)-based delivery approaches for the transfection of CRISPR reagents, while representing a promising association as will be addressed later in this review, toxicity concerns have been raised (Demirer et al., 2021). As such, systemic toxicity studies have suggested that the physical and chemical properties of nanomaterials must be taken into account. For example, in the case of the carbon nanotube, in which limitations of its use have been emphasized due to the non-biodegradable nature and the presence of heavy-metal impurities introduced during NPs synthesis (Kostarelos, 2008; Pikula et al., 2020). In this setting, to avoid future problems in the United States, the application of new substances as nanocarriers in agriculture must demonstrate safety and absence of toxicity effects before its application in the field, following the regulation of the Toxic Substances Control Act (TSCA) law (Heller et al., 2020). Furthermore, the generation of data about nanomaterial’s lifecycle in CRISPR/Cas edited plants and their progeny, its fate in the environment, likewise the potential impacts on interacting organisms, including humans, may provide crucial information towards the approval of new, safer, and more sustainable NPs (Demirer et al., 2021).

### 2.3 Strategies to Increase On-Target Specificity/Efficiency and Avoid Toxicity in Plants

Efforts have been made toward the optimization of CRISPR/Cas strategies to increase on-target specificity/efficiency as well as reduce off-target effects and toxicity in plants (Hajiahmadi et al., 2019). In general, the main technical factors that may influence undesirable outcomes in plants are the gRNA design, choice of Cas variant proteins, specific CRISPR component formats, and the delivery methods of CRISPR/Cas reagents into the target genome.

#### 2.3.1 Properties and gRNA Design

A prerequisite for reducing off-target effects is optimizing the gRNA design, and carefully selecting the sequence to be targeted (Hsu et al., 2013; Zhu et al., 2017; Zischewski et al., 2017). Bioinformatics web-based tools have been developed for the gRNAs design and to predict potential off-target sites in plant genomes, including Cas- OFFinder (Bae et al., 2014), CHOPCHOP v.2 (Labun et al., 2016), CRISPOR (Haeussler et al., 2016), CRISPR-P 2.0 (Liu et al., 2017), CRISPR-GE (Xie et al., 2017), CRISPR-PLANT v.2 (Minkenberg et al., 2019), and CRISPR-BETS (Wu et al., 2022). For more details, refer to Gerashchenkova et al. (Gerashchenkova et al., 2020), which describes over a hundred software for gRNAs design. Hahn and Nekrasov (Hahn and Nekrasov, 2019) emphasize that the species having annotated genome sequence available is not mandatory, but necessary for an effective prediction once it would allow examining off-targets also located in non-coding regions. In summary, these above-mentioned tools consider incompatibilities within the gRNA seed sequence (8–12 nucleotides upstream to PAM), being its number and position decisive for gene editing specificity. In addition, mismatches located between the eight nucleotides proximal to the PAM site reduce off-target effects. According to Modrzejewsk and co-workers, the off-target effect rate decreases 59% if there is a unique mismatch between the target and off-target sequence. In the cases that there are four or more mismatches, this value reduces further to 0.09% (Modrzejewski et al., 2020).

Another factor to consider for enhancing gRNA specificity is the ratio of guanine-cytosine (GC) nucleobases, even though there is no consensus among the studies. The hypothesis is that a low GC content decreases off-target occurrence (Yu et al., 2017), as the high content stabilizes the hybridization of gRNA to genomic DNA (Fu et al., 2013). While some studies have shown that gRNA sequences with low (<20%) or high CG (>80%) content are less effective against targets (Wang et al., 2014b; Ma et al., 2015), others did not identify interference from the total GC content of gRNA (Ren et al., 2014; Jensen et al., 2017; Labuhn et al., 2018; Modrzejewski et al., 2020). Recently, the study by Malik et al. (Malik et al., 2021) showed that the high GC content in the seed region (1–12 nucleotides close to PAM) decreases the activity of gRNAs, negatively influencing the target cleavage efficiency. So, the use of intermediate GC contents (∼50%) is indicated as a reference for gRNA design to improve the on-target specificity. However, more studies are needed to better elucidate how it operates.

#### 2.3.2 Cas Protein Variants

Limitations for CRISPR technology using *Sp*Cas9 include protein size, off-target effects, and the requirement of a specific PAM sequence (NGG) in the genome, which restrain potential target recognition sites (Zhi et al., 2021). Two main approaches have been adopted as alternatives to overcome this restriction: the use of Cas9 orthologs derived from different organisms and the Cas9 protein modification to recognize different PAM sequences (Sukegawa et al., 2021). For a full list describing natural and engineered Cas nuclease variants used in genomic editing, refer to Anzalone et al. (Anzalone et al., 2020).

Natural Cas9 variants presenting different PAM sequences, such as those from *Staphylococcus aureus* (*Sa*Cas9—NNGRRT), *S. thermophilus* (*St1*Cas9—NNAGAAW, W = A/T), and *S. canis* (*Sc*Cas9—NNG) had their specific recognition sites demonstrated in different plant experiments (Steinert et al., 2015; Kaya et al., 2016; Wang M. et al., 2020; Veillet et al., 2020). Numerous engineered variants have also been developed (*Sp*Cas9-VQR, *Sp*Cas9-EQR, *Sp*Cas9-VRER, *Sp*Cas9-NG, *Sp*Cas9-HF1, *eSp*Cas9, *Hypa*Cas9, *evo*Cas9, *Sniper-*Cas9, *x*Cas9, and *Sp*RY) based on the crystal structure of Cas9 attached to gRNA and target DNA (Wada et al., 2020). These natural and engineered variants exhibit relaxed PAM sites, smaller sizes compared to *Sp*Cas9 (1,368 aa), high target specificity, and promising applications (Negishi et al., 2019; Qin et al., 2019; Xu et al., 2019; Zhong et al., 2019).

The nuclease Cas12a (previously called Cpf1), widely used for genome editing in plants, opened the possibility to target adenine-thymine-rich genomic regions (Zetsche et al., 2015). Cas12a has a PAM sequence rich in “T” nucleotides (TTTV, V = A/G/C). Unlike Cas9, Cas12a has two RuvC catalytic sites, its cut generates blunt ends in the DNA double-strand, and it does not have tracrRNA (trans-activating CRISPR RNA) in the system. These properties make this nuclease more suitable for generating larger deletions and multiplex gene editing (Zhang et al., 2021b; Huang Holger Puchta et al., 2021).

The dCas9 engineered variant enzyme is able to alter the phenotype (e.g., modulating gene expression and/or translation) without changing the genetic code of plants, thus representing an interesting alternative approach to reduce off-target effects, bypass DSB-induced toxicity, avoiding pleiotropic and lethal effects in the targeted plant (Lei et al., 2013; Brezgin et al., 2019). The use of two dCas9 simultaneously at the same locus to cleave each DNA strand has also been proposed to reduce potential off-target effects (Pereira, 2016).

#### 2.3.3 Alternative CRISPR Component Formats

In general, plasmid DNA expression vectors harboring a CRISPR gene cassette are used in the genetic transformation of target organisms via *Agrobacterium tumefaciens* (Das et al., 2021) or through particle bombardment (Imai et al., 2020). However, this most frequently applied strategy has as major concerns the random integration into the genome and the continuous expression of Cas protein and gRNA(s), which increases the possibility of chimeric mutants, off-target effects, and toxicity (Feng et al., 2014; Hashimoto et al., 2016). To overcome these issues, the availability of different CRISPR/Cas system reagent formats, such as mRNA and pre-assembled RNPs, represent promising alternatives (Liang et al., 2017). RNP-based DNA-free genome editing in plant cells usually occurs through PEG, electroporation, lipofection, and particle bombardment (Zhang et al., 2021a). After delivering the complex into the cell nucleus, RNP is rapidly degraded, thus avoiding potential off-target effects (Kim J.-S. et al., 2015, 2017; Subburaj et al., 2016). Moreover, for cellular toxicity associated with long-term expression of Cas and/or integration of exogenous DNA, the RNP complex approach may represent a good choice due to the transient and stable transfection in the plant cell (González et al., 2021). On the other hand, as this strategy does not use selection marker genes, the screening of edited plants with desirable phenotypes may become more laborious and costly. Additionally, this method often presents lower editing efficiencies (∼10%) compared to stable integration vectors, as already demonstrated for corn (*Zea mays*) (≤9.7%) (Svitashev et al., 2015), brassica plant species (≤24.51%) (Murovec et al., 2018), potato (*Solanum tuberosum*) (≤25%) (Andersson et al., 2018), and petunia (*Petunia juss*) (≤11.9%) (Yu et al., 2021).

#### 2.3.4 The Use of Viral Vectors and the Association With Nanomaterials

The success of CRISPR technology relies directly on the approach used to deliver its reagents. However, the cargo of biomolecules consists of one of the main steps and bottlenecks in genetic transformation. Unlike animals, plant cells possess a cell wall that represents a natural physical barrier limiting the entrance of exogenous molecules into the cytoplasm. Biolistics and *A. tumefaciens* transformation are the conventional methods typically used to overcome plant cell wall, but these approaches have several disadvantages that can negatively impact the transformation process, such as low efficiency of target edition, plant tissue damages, and technical incompatibilities (Altpeter et al., 2016; Demirer et al., 2021). Notwithstanding, the recent advancements in the field of delivery using viral vectors and nanoparticles have delineated new possibilities, surpassing traditional limitations and contributing to improvements in the genetic engineering of plants (Cunningham et al., 2018).

Some viruses are efficient in the genomic editing of plants due to their ability to infect and replicate into the cells of a wide range of plant species (Zhu et al., 2020). Over the years, there have been remarkable advances in virus research as carrier agents involved in plant genome editing, also known as virus-induced genome editing (VIGE) (Gentzel et al., 2022). In principle, only engineered RNA or single-stranded DNA viruses positive-sense were used to express gRNA strands, however requiring the expression of Cas protein in genetically modified plants for the effectiveness of the CRISPR/Cas system (Ali et al., 2015a, 2015b; Yin et al., 2015; Oh et al., 2021). Later, it is possible to stably express Cas and gRNA in single-stranded RNA virus negative-sense. For example, *Barley yellow striate mosaic virus* (BYSMV) and *Sonchus yellow net virus* (SYNV) were able to efficiently edit *Nicotiana benthamiana*, but without transgenerational effect due to the inability of these viruses to penetrate the meristematic and reproductive tissues of the plant (Gao et al., 2019; Ma and Li, 2020). Currently, the fusion of mobile elements to the gRNA in the infection clone has belonged to the presence of the virus in meristematic tissue, consequently inducing the mutation in the progenies (Ellison et al., 2021; Lei et al., 2021).

In theory, all available formats of CRISPR/Cas system reagents (e.g., plasmid DNA expression vectors, mRNA, and RNP complexes) can be encapsulated in nanomaterials prior to cell delivery. Nanomaterials can improve cellular uptake, as well as circumvent technical limitations, such as the low stability of CRISPR reagents depending on the format chosen (Demirer et al., 2021). A multitude of NPs have been developed and tested in an attempt to improve the transformation efficiency of different crops, however few of them have been successful as carriers of CRISPR/Cas system components. The most common nanomaterial tested to deliver DNA and other chemical reagents in plant cells is mesoporous silica (Torney et al., 2007). Other well-known NPs are carbon nanotubes, which are passively absorbed by plant cells without being degraded by endonucleases (He and Zhao, 2019). Promising results using these NPs to nanoencapsulation and deliver plasmid DNA into chloroplast organelles have been reported in brassica, cotton (*Gossypium hirsutum*), and wheat (*Triticum aestivum*) (Kwak et al., 2019; Demirer et al., 2020). Likewise, layered double hydroxides (LDHs) and carbon dots are also a good choice of NPs, once they can penetrate plant cells causing minor injuries and efficiently protecting the internalized content (Bao et al., 2017). Doyle et al. (Doyle et al., 2019) performed one of the few studies in the literature reporting the use of NP to deliver CRISPR/Cas components to plant cells. Authors showed that naturally occurring carbon dots (quasi-spherical, <10 nm nanoparticles) can be used as a vehicle for carrying Cas9 and gRNA plasmid coated carbon dots into wheat plants via foliar application by spraying and to generate target mutations. Instead, Sandhya et al. (Sandhya et al., 2020) suggest the direct delivery of RNPs to regenerative tissues using a pollen magnetofection-mediated delivery. The methodology aims to use pollen as a nanocarrier agent for exogenous DNA molecules, and later the use of this pollen to fertilize the plant’s ovary and directly induce the genetic edition of seeds.

Altogether, the rapid evolution of CRISPR/Cas technology and all associated-approaches/strategies available for plant genome editing provide optimal conditions to target the above-mentioned technical-related challenges and also to improve the understanding of risk/safety implications. Lastly, although concerns about unintended off-target effects and potential toxicity have raised discussions around CRISPR adoption in plant breeding, these should not be considered criteria for restricting CRISPR technology application, as in the case of its usage in animal cells, apparently. Moreover, in most plant species it is possible to eliminate off-target mutations and inferior traits through genetic segregation by the backcross breeding approach (Murovec et al., 2018).

## 3 RNAi Plant-Based Technologies

RNAi-based transgenic plants, designed to express dsRNA sequences to knock down the expression of specific genes in the host and/or pathogen genome, have represented a remarkable complementary tool to face the abusive usage of pesticides in agricultural fields, with great potential to cause environmental and human health problems (Mezzetti et al., 2020). However, in the last few years, the global demand for a more sustainable and non-transformative technologies of crop protection has substantially intensified (Budzinski and Couderchet, 2018; Fletcher et al., 2020). In this context, the scientific community has strived to develop and master the application of novel non-transgenic RNAi-based technologies.

### 3.1 Topical RNAi-Based Approach Towards a More Sustainable Plant Protection

The breakthrough and Nobel Prize-winning discovery that oral delivery of dsRNA to *C. elegans* induced a potent and specific gene silencing (Fire et al., 1998), nourished the perception that exogenous dsRNA application could trigger RNAi response on any target organism, and paved the way for the emergence of the topical RNAi-based technology. Such approach consists of producing high amounts of self-delivering dsRNAs to be topically used in the field as bio-defensive molecules (Das and Sherif, 2020), so far standing as a promising tool in agriculture to achieve plant protection against several pathogens (Dalakouras et al., 2020; Kiselev et al., 2022).

Tenllado and Diaz-Ruiz (Tenllado et al., 2003) and Tenllado et al. (Tenllado et al., 2003) were the first to report the plant protection from viruses by topical dsRNA application. They showed the foliar application of *in vitro* expressed dsRNA molecules targeting the plant viruses *Pepper mild mottle virus* (PMMoV), *Plum pox virus* (PPV), *Alfalfa mosaic virus* (AMV), and *Tobacco etch virus* (TEV) conferred plant resistance against infections. Following this pioneering discovery, different studies reported successful control of multiple families of plant viruses by topical RNAi-based technology (Mitter et al., 2017).

Similar to viruses, fungal control by topical application of dsRNAs seems to be promising. Koch et al. (Koch et al., 2013) showed that *in vitro* cultures of *Fusarium graminearum* treated with dsRNAs complementary to three cytochromes P450 (CYP) genes resulted in growth inhibition, similarly to the observed after treatment with fungicide tebuconazole. Also, they reported that topical application of these dsRNAs on detached barley leaves impaired *F. graminearum* growth beyond the applied sites, suggesting a systemic activity (Koch et al., 2016). Moreover, the surface of fruits, vegetables, and flowers sprayed with dsRNAs targeting two DICER-LIKE (DCL) genes of *Botrytis cinerea* resulted in effective control of the pathogen, demonstrating that topical RNAi-based approaches may be useful to protect crops either during the production cycle as in post-harvest stages (Wang et al., 2016).

The first demonstration of exogenous dsRNA application against insect pests came from a study on citrus and grapevines to control two hemipteran pests, the xylem-feeding glassy-winged sharpshooter *Homalodisca vitripennis*, and the phloem-feeding psyllid *Diaphorina citri*. Both insects tested positive for dsRNA ingestion after feeding on plants treated with dsRNAs applied to the root zone, showing the movement of dsRNA through the graft junction of rootstock and scion (Hunter et al., 2012). Later, San Miguel and Scott (San Miguel and Scott, 2016) demonstrated the dsRNA application on leaves of potato plants targeting the actin gene on Colorado potato beetle (*Leptinotarsa decemlineata*) resulted in significant mortality of insects. Moreover, the result showed that dsRNA remained biologically active on potato leaves for at least 4 weeks under greenhouse conditions.

Although mounting evidence demonstrates the efficacy of topical RNAi-based technology to enhance quantitative and qualitative valuable agronomic crop traits, relevant concerns have been raised about its feasibility, from delivering methods to relative costs of the technology, as well as the associated risks. All these matters must be overwhelmed to allow a straightforward translation of research data into new biotechnological commercial solutions, intending to minimize environmental, health, and regulatory issues.

### 3.2 Risks and Challenges Involving Topical Application of dsRNA

Topical RNAi-based technologies offer clear benefits over most existing crop protection chemical pesticides. However, an approach based on scientific parameters to develop and validate procedures is fundamental to defining which risk assessment criteria are most appropriate for these technologies (Mezzetti et al., 2020).

#### 3.2.1 Weighting the Unintended Off-Target Effects

Usually, off-target effects are due to the existence of any degree of sequence similarity between siRNA (e.g., synthetic and/or derived from dsRNA processing by DICER enzyme) and non-target mRNA transcripts (Chen et al., 2021a). In this context, the presence and position of nucleotide mismatches along with the siRNA molecule structure, in relation to the target sequence, seem to exert a major influence in the silencing of nontarget genes. Kulkarni et al. (Kulkarni et al., 2006) investigated dsRNA specificity using the model insect *Drosophila melanogaster*. Through a high-throughput screening, authors reported that long dsRNAs sharing a perfect identity of as few as 19 nt-long with predicted unintended targets, lead to off-target effects. Investigating governing rules of dsRNA specificity in the beetle *Tribolium castaneum*, Chen et al. (Chen et al., 2021b) showed that a dsRNA targeting a member of the CYP, the gene CYP6BQ6, was able to silence another eight genomic regions with nucleotide sequence identity ≥68%. Among these genes, CYP6BK7 and CYP6BK13 showed significant alteration in transcript modulation. Sequence analysis found that CYP6BK7 and CYP6BK13 contain 24 and 26 bp of contiguous matching bases with only two single mismatched bases, respectively. Further investigations using mutational analyses showed that dsRNAs with ≥16 bp perfectly matched sequence or >26 bp almost perfectly matched sequence (i.e., with one or two mismatches scarcely distributed) were also able to trigger RNAi gene silencing on *T. castaneum* off-target transcripts. Taning et al. (Taning et al., 2021a) used a sequence complementarity-based approach to evaluate potential off-target effects in bumblebee (genus *Bombus*), following oral exposure to a chimeric dsRNA. Interestingly, no modulation was found in the transcript level for all potential predicted off-targets, including sequences with 20 continuous nucleotide matches or with 21 bp stretch with only one mismatch.

Besides the important role of nucleotide mismatches, as well as the apparent variation in the occurrence of off-target gene silencing between organisms, two other ways may trigger off-target activity. First, the RNAi enzymatic complex (more specifically the Argonaute RISC Catalytic Component 2- AGO2 enzyme) can erroneously incorporate the wrong strand of siRNA sequence (e.g., the passenger strand) leading to the downstream degradation of unintended transcripts (Schwarz et al., 2003). The second and unpredictable triggering of off-target activities may occur if the small RNA binds to the miRNA pathway, which can result in the silencing of dozens if not hundreds of transcripts (Doench et al., 2003; Jackson et al., 2003).

#### 3.2.2 Cross-Kingdom Nontarget Risks and Related Biosafety Issues

Considering that off-target effects are usually surveyed only within target organisms, very little is known about how dsRNAs affect the gene silencing in nontarget organisms. Suffice to say that, aiming at the generation of RNAi-based technological solutions for agriculture, the risk analysis should encompass each of the myriad interacting organisms in the agroecosystem, including humans, that may be directly or indirectly exposed to the dsRNA molecules.

The fact is that cross-species and cross-kingdom nontarget effects may occur more often than is commonly argued. For example, Zhang et al. (Zhang et al., 2011) reported a quite intriguing result showing that a great amount (up to 10%) of plant exogenous microRNAs were found in sera and tissue samples of various animals and that these are likely taken orally with food. MIR168a is a plant miRNA very abundant in rice crops. Surprisingly, the amount of MIR168a was found to increase in the serum of rats fed with a rice-containing diet, even when it was cooked. Following *in vitro* and *in vivo* functional assays showed the ability of rice MIR168a to bind both to human and mouse transcripts, resulting in non-target gene silencing effects. Another interesting study demonstrated that dsRNAs expressed by transgenic maize crops, and designed to silence target genes in the western corn rootworm (Baum et al., 2007). *Diabrotica virgifera* also impacted the expression of orthologous gene members present in the other insect species, *D. undecimpunctata* and *L. decemlineata*, despite the relatively low sequence homology between genes in the target and nontarget organisms.

In terms of cross-kingdom dsRNA transference, recent studies have shown the transference of small RNAs between plants and pathogens occurs spontaneously in nature, participating mainly in defense mechanisms (Guo et al., 2018). For example, cotton plants produce miRNAs (e.g., miR166 and miR159) which are exported directly to the hyphae of the fungus *Verticillium dahliae*, a vascular pathogen responsible for wilt in many cotton crops, targeting transcripts engaged with fungus’s virulence, and conferring plant resistance (Zhang et al., 2016). Likewise, it was described that small RNAs (e.g., TAS1c-siR483 and TAS2-siR453) produced by the model plant *A. thaliana* were detected in cells of the fungus *B. cinerea* during plant-pathogen interaction, and plant lines overexpressing these small RNAs displayed reduced susceptibility to this pathogen, which showed a negative modulation of targeted transcripts (Cai et al., 2018).

Similarly, small RNAs can also be transmitted in the opposite direction, i.e., from the pathogen to the host plant. One of the first studies that demonstrated the transfer of small RNAs from pathogens to plants was performed by Weiberg et al. (Weiberg et al., 2013), where three siRNAs from the fungus *B. cinerea* (Bc-siR3.1, Bc-siR3.2, and Bc-siR5), with predicted targets in *A. thaliana* and tomato (*Solanum lycopersicum*) plants, rendered both host plants susceptible to fungus infection. Furthermore, *Arabidopsis AGO1* mutants, unable to process the small RNAs from *B. cinerea*, exhibited reduced susceptibility to the fungus, as well as to *B. cinerea* DCL1/DCL2 double mutant, which exhibited reduced pathogenicity on both plants.

The natural traffic and delivery of these small RNA molecules inside and between interacting plant-pathogen organisms can be done through extracellular vesicles (EVs), i.e., membrane-bound particles that carry manly transmembrane proteins and RNAs, being produced by both sides of the pathosystem (Liu et al., 2021). In plants, stress-associated EVs were isolated and characterized in apoplast fluids from *Arabidopsis* leaves, from where they are assimilated by the pathogen/pest (Rutter and Innes, 2017). Different studies on the *A. thaliana* and *B. cinerea* interaction have demonstrated the transfer from plant to fungus of “tiny RNAs,” which are 10–17 nucleotides in length, and derived mainly from the positive strand of mRNA transcripts (Cai et al., 2018; Baldrich et al., 2019). On the other direction, recent studies have also reported the EVs delivery from pathogens to plants. Bleackley et al. (Bleackley et al., 2020) demonstrated that EVs secreted by the fungus *F. oxysporum* induced phytotoxic responses in cotton plants. Likewise for the fungus *Zimoseptoria tritici*, whose EVs are engaged with the triggering of pathogenesis in wheat crops (Hill and Solomon, 2020).

Based on the studies of cross-kingdom small RNA transfer, small RNAs exchanged between plants and pathogens could have five possible fates: 1) if the expression is not sufficient and the concentration of small RNAs is low, the transferred dsRNA could be diluted during proliferation and division of the recipient cell; 2) RNAi-mediated signaling can be amplified by the production of secondary siRNAs; 3) the transferred RNAs can be degraded by RNAi suppressor proteins; 4) long dsRNAs can activate RNAi system and induce gene silencing in recipient plant and; 5) the transferred RNAs can improve the adaptability of recipient plants to the environment and it can be retained and fixed in the genome of the recipient plant through horizontal gene transfer (Zhao et al., 2021).

#### 3.2.3 Challenges Related to the Uptake and Stability of Topically-Applied dsRNA

The advantages of topically-applied dsRNA and its potential as a biopesticide commercial product are still hindered by technical issues, including molecule uptake and stability, delivery methods, inconsistent activity of the dsRNA trigger, and activity level of RNAi suppression (Hunter et al., 2021). Hence, one of the first aspects that should be addressed when thinking about topical RNAi-based technology is the uptake efficiency of dsRNAs and/or siRNAs/miRNAs either by the pathogen or plants, depending on the adopted strategy. In the case of having phytonematodes as a target for gene silencing, self-delivering dsRNA molecules are ideally supplied as food nearby the plant root *in vivo* assay and the uptake is made by pathogen’s ingestion. Once in the midgut cells of the nematode, the molecule internalization is mediated by several transmembrane proteins, known as systemic RNA interference deficiency (SID), in particular, the proteins SID-1 and SID-2, triggering a systemic gene silencing (Winston et al., 2002; Wang and Hunter, 2017; Whangbo et al., 2017). For insects, there are two described mechanisms of dsRNA uptake: 1) likewise mediated by SID-like proteins (SIL), and 2) clathrin-mediated classical endocytosis. Different studies on the dsRNA uptake by *Apis mellifera* (Aronstein et al., 2015), *D. virgifera* (Miyata et al., 2014), and *L. decemlineata* (Cappelle et al., 2016) reported that either the overexpression or knockdown of SIL genes caused variations in gene silencing, while in *Plutella xylostella* (Wang et al., 2014a), *Schistocerca gregaria* (Wynant et al., 2014) and *Tribolium castaneum* (Tomoyasu et al., 2008), the knockdown of those genes did not affect uptake efficiency (Xu and Han, 2008; Bansal and Michel, 2013). Moreover, the molecular process generally involves the recognition of dsRNAs by scavenger receptors, which can be influenced by the length of dsRNA molecules, as demonstrated for species of the order Coleoptera, where very small dsRNAs were not effectively internalized (Miller et al., 2012). Although these represent the most accepted and well-described models for dsRNA uptake in insects, it is still not known why dsRNA remains within the endosomes of some species of the order Lepidoptera, which directly influences the efficiency of RNAi-mediated gene silencing (Yoon et al., 2017). For other disease-causing agents of plants, such as phytopathogenic fungi, the knowledge about dsRNA uptake mechanisms is still limited. However, a recent study with *Sclerotinia sclerotiorum* suggested that dsRNA uptake is mediated via classical clathrin endocytosis, likewise for insects, but dsRNA recognition receptors remain elusive (Wytinck et al., 2020).

Ultimately, it is reasonable that dsRNA uptake efficacy may vary due to numerous factors, including differences in insect feeding behavior, its availability on feeding sites, lack of gene silencing amplification signal, and also dsRNA degradation during ingestion (Niu et al., 2019). Furthermore, even though *in vitro* assays involving the oral feeding of pathogen and/or disease-carrying insect vectors with dsRNAs targeting their essential genes have been shown to induce consistently high mortality, reproducing these results on the field conditions by topical application strategy represents a great challenge.

Another approach to improve plant crop resistance consists in the foliar application of self-delivering dsRNAs to the plant surface, aiming at the knockdown of plant genes whose expression is associated with pathogen susceptibility. Such strategy is likewise crucial to ensure that topically-applied dsRNA display both appropriate stability, to hinder dsRNA premature degradation by environmental factors (e.g., rainwater, sunlight/UV radiation, and microorganisms), and a great capacity to penetrate the natural plant foliar barriers, such as waxy cuticles, trichomes, and the cell wall (Bennett et al., 2020; Rank and Koch, 2021). Therefore, due to these significant challenges, there are still very few studies reporting the success of this approach. Dubrovina and co-workers (Dubrovina and Kiselev, 2019) showed that a prior foliar cuticle abrasion through a high pressure using microparticles may facilitate dsRNA absorption by plant cells. Likewise, the use of surfactant agents has been shown to improve dsRNA entrance through the foliar stomatal aperture (Bennett et al., 2020).

Furthermore, studies have shown that dsRNA molecules are degraded very rapidly in the environment (Bachman et al., 2020). Therefore, another point to be addressed is the increase in dsRNA protection window, which is very short when applied “naked,” limited to a few days in the environment (Rego-Machado et al., 2020). In the case of topical RNAi-based products, in which dsRNAs may be conjugated with nanoformulations to increase their absorption, and stability, among other parameters, a case-by-case risk assessment should be required (Mendelsohn et al., 2020).

### 3.3 Strategies to Increase On-Target Specificity, Stability, and Delivery of Exogenous dsRNA

#### 3.3.1 dsRNA Molecule Design

The accumulated experimental data is helping to increase the accuracy of prediction models and RNAi design tools, which allows inferences about the efficiency of the dsRNA *in silico*. To obtain the greatest efficiency of the RNAi technology, three factors must be taken into account: 1) the number of siRNA generated from a single dsRNA; 2) the specificity of the siRNA to the target transcript, and 3) chemical alteration in the seed region of the siRNA guide strand. The enzyme DICER endonuclease attaches to longer dsRNAs, resulting in the accurate cleavage of dsRNAs into shorter siRNAs. The presence of the DICER cleavage site increased effectiveness up to 100-fold compared to a sequence without the site (Cooper et al., 2021). It has also been proposed that apart from the cleavage of longer dsRNAs, DICER endonuclease plays important role in the loading of cleaved dsRNA into the RISC complex (Lee et al., 2004; Vergani-Junior et al., 2021). Thus, the presence of DICER enzyme sites is desirable when selecting target regions for dsRNA design. Another interesting optimization of dsRNA molecule aiming at enhancing dsRNA activity for exogenously applied treatments to plants and insect ingestion was demonstrated by Hunter and Wintermantel (Hunter et al., 2021). Authors reported that chemically-modified dsRNAs incorporating 2′-F pyrimidine nucleotides (32–55%) along with dsRNA structure, led to considerable improvements in the RNAi activity across multiple Hemipteran insect plant-disease vectors which reflected in increased insect mortality by 12–35% greater than non-modified dsRNAs displaying the same sequence.

Fortunately, the availability of stringent software to design dsRNAs has largely minimized the occurrence of off-target and nontarget effects by predicting the degree of sequence homology between the antisense strand of siRNAs and target transcripts (Knott et al., 2014; Lück et al., 2019). However, for species lacking genome/transcriptome sequence annotation on databases, such bioinformatic-based dsRNA design may require alternative tools and even more important, supplemental information about the biology of target organisms and the existing ecological interactions, in which the dsRNA will be applied (Fletcher et al., 2020).

#### 3.3.2 dsRNA Association With Nanomaterials

A promising alternative to circumvent all aforementioned constraints mainly related to dsRNA uptake, delivery, and stability, boosting the practical use of topical RNAi-based technologies, is the association with nanobiotechnology. The nanomaterial can be engineered to synthesize NP that operates as nanocarriers for the delivery of dsRNAs, providing several advantages, including protection/stability enhancement of dsRNA molecules, improvement of foliar/microorganism surface adherence, and cell internalization, with positive impacts on the efficacy of RNAi gene silence response (Ghormade et al., 2011; Adeyinka et al., 2020). There is an ever-expanding list of NPs that have already been tested as dsRNA carriers, and they are usually made from lipid biomolecules or different polymers, which can be natural (e.g., agar, starches, alginates, chitosan, and cellulose), synthetics [e.g. poly(vinyl alcohol)—PVA, poly(ethylene glycol)—PEG, and poly(lactic-co-glycolic acid)—PLGA] or hybrids (Sikder et al., 2021). The major challenge in elaborating these NPs lies in the fact that they need to be quite stable, non-toxic, eco-friendly (e.g., biodegradable), and easy to be conjugated with RNAs molecules. Moreover, there are several relevant characteristics of the NPs to be taken into account for the efficient delivery of dsRNAs. For example, in theory, particles larger than 5–20 nm are not capable of entering the plant cell wall (Schwab et al., 2016). Likewise, NPs must be designed to carry positive amino groups to allow the binding with the negatively charged dsRNAs phosphate groups (Avila et al., 2018). Lastly, the complex NP-dsRNA must be able to dissociate into the cell cytosol, and the addition of polyanions molecules or acid solution can confer such ability (Yan et al., 2021).

Among the dsRNA nanoformulations, lipid-based NP (e.g., liposomes and micelles) and chitosan-based dsRNA formulations are by far the most widely used nanocarriers. Numerous studies mostly involving insect species (e.g., *Aedes aegypti*, *Blattella germanica*, *Chilo suppressalis*, *D. melanogaster*, *Euschistus heros*, *Ostrinia nubilalis,* and *Spodoptera frugiperda*), have reported success using these NPs carrying small RNAs to knockdown different gene targets, showing as well an enhancement of dsRNA stability in the presence insect endonuclease enzymes (Wang K. et al., 2020; Christiaens et al., 2020; Gurusamy et al., 2020; Cooper et al., 2021). However, although the high efficiency of lipid-based vesicles in the control of plant pathogens/pests, its practical usage is majorly halted by the high cost and dependence of adjuvants (e.g., surfactant, emulsifier, and stabilizer) used on the generation process (Bauer et al., 2006; Azarnezhad et al., 2020). Nevertheless, several other innovative NPs have been created, expanding dsRNA delivery strategies. Mitter et al. (Mitter et al., 2017) complexed dsRNA with LDH nanosheets, termed Bioclay, which allowed to expand the window of protection from viral pathogens from 5 to 7 days to more than 20 days. Another formulation complexing NP-dsRNA-adjuvants was able to penetrate through the aphid body wall into the haemocoel and spread into various tissues, resulting in significant knockdown of target gene expression and insect mortality (Zheng et al., 2019). Even in recalcitrant insects such Lepidoptera, dsRNA complexed with a synthetic cationic polymer, poly-[N-(3-guanidinopropyl)-methacrylamide], was effectively taken up by *S. frugiperda*, resulting in significant knockdown and larvae mortality (Parsons et al., 2018).

Furthermore, complexing dsRNA molecules to NP hold also the potential to address another big challenge related to the cost of dsRNA synthesis. The production of quantity and quality dsRNA for spray applications is still considered expensive, although the cost (per Gram) to synthesize dsRNA has been considerably reduced, dropping from U$ 12.500 in 2008 to U$ 0.5 in 2021 (Zotti et al., 2018; Rank and Koch, 2021). A low-cost dsRNA production is imperative due to the necessity of applying approximately 2–10 g of dsRNA per hectare (Zotti et al., 2018).

Taken together, technological advances in dsRNA nanoformulations hold the capacity to overcome inherent bottlenecks of topical RNAi-based technique, providing the desirable molecule protection, higher efficiency of dsRNA uptake, and delivery, beyond reducing potential collateral environmental risks. All crucial features for the full establishment of these next-generation crop protection solutions.

## 4 Public Acceptance and Regulatory Aspects of CRISPR/Cas and Topical RNAi-Based Technologies

The fact that only a handful of these bioproducts and varieties have been approved for commercial release worldwide is probably not only related to the everlasting regulatory hurdles, but also unsettled consumer perception and acceptance (Mat Jalaluddin et al., 2019). According to Taning et al. (Taning et al., 2021b), for society to accept biotechnology products, diverse key tasks should be addressed.

In terms of reporting biotechnology advancements, regular communication among researchers, farmers, and other relevant players in the food production chain are crucial to reassure stakeholders, assist regulatory compliance, and also to support the general public (e.g., consumers) perception. Moreover, the public acceptance of CRISPR/Cas- and RNAi-based bioproducts (e.g., plant crop resistant varieties, biopesticides), mostly relies on a proper and unbiased broadcast addressing technical issues (e.g., gene editing/silencing driving mechanisms), as well as all potential negative and positive (risk-benefits) related impacts. In this process, scientists may play key roles in finding instruments for a straight dialogue with civil society organizations, and supporting educational initiatives (Taning et al., 2021b; Rank and Koch, 2021).

Ethical and moral issues should also be properly addressed early on in the development process of CRISPR/Cas- and RNAi-based technological solutions, since these concepts exert a strong appeal to the target audience (Frewer et al., 2013; Gupta et al., 2015). According to Beghin and Gustafson (Beghin and Gustafson, 2021), most consumers are willing to consume and pay for foods derived from more sustainable plant engineering techniques, especially if they embody useful traits for the environment, animal, and human health. Additional studies have suggested that the use of topical applied RNAi-based products for plant crop disease management may increase public acceptance since this new technology does not involve a stable expression of transgenic genetic elements by treated organisms (Shew et al., 2017). Similar public behavior was observed for the food consumption of non-transgenic CRISPR/Cas bioproducts already launched (Shew et al., 2018). In these two last-mentioned studies, the authors aimed to test the market viability of RNAi- and CRISPR-based bioproducts, respectively. For this purpose, consumers from different countries, including the United States, Canada, Australia, France, and Belgium, were surveyed for their preference for consuming three bioproducts: a hypothetical GMO rice variety developed by using *Bacillus thuringiensis* (Bt) transgene technology, a hypothetical non-GMO rice variety generated by SIGS approach (i.e., topical RNAi-based technology), and a CRISPR-based crop. The results showed that applicants from all countries were far more inclined to consume non-GMO rice. In addition, authors reported that on average, half of the participants would consume both GMO and CRISPR food. Further studies and more exhaustive field surveys are very welcomed to endorse public acceptance and perception of these new agricultural technologies. Ultimately, to assure a sustainable production of high-quality food, the entire production chain must be ruled with parsimony and balance between environmental, economic, and social claims, as well as be assisted ideally by strong public policies that safeguard consumers’ health and their concerns (Montenegro, 2016; Hamburger, 2018).

Concerning the regulatory aspects of CRISPR edited plants, even though the discussion is still ongoing worldwide, several countries already have specific regulatory policies for evaluating these products. Technologies generated through gene editing can be classified as SDN1, SDN2, and SDN3 (SDN, site-directed nucleases) following the terminology proposed by Podevin et al. (Podevin et al., 2013). Regarding the SDN1 strategy, the non-homologous end joining (NHEJ) cell repair pathway is explored mainly to induce gene knockout. In the case of SDN2, the homology-directed repair (HDR) pathway is used to introduce mutations resulting in the alteration of one or few base pairs, for example, to make an allelic substitution. In the SDN3, although it explores the same repair pathway as in SDN2, the inserted sequence is longer and could be a promoter, coding, or terminator region, from a sexually compatible species or not (Podevin et al., 2013). Therefore, depending on the strategy employed, it could or not generate a final product ruled as GMO, even though in many cases it does not involve the introduction of exogenous DNA sequences.

The worldwide scenario of regulatory policies for the evaluation of CRISPR edited plants is changing rapidly and continues to evolve as more countries launch their own regulatory policies, an expanding list which includes so far Argentina, Brazil, Chile, Colombia, United States, Paraguay, Japan, Australia and, more recently, the United Kingdom (Entine et al., 2021). The main focus of the deliberations is still on the question of “*be or not to be*” a GMO and, although the criteria adopted by each of these countries are quite different, in most situations the risk assessment is evaluated case-by-case. Such tailored-made assessment takes into account specific parameters, including the CRISPR-toolbox strategy employed for genome editing, the resulting combination of the genetic material, whether the mutation could be generated by conventional breeding or mutagenesis, and the absence of recombinant DNA in the final product (Molinari et al., 2021).

Briefly, according to the aforementioned legislation, mutations produced by SDN1 systems generate products not qualified as GMOs, and for this reason, they are not evaluated under the same criteria applied for conventional genetically modified products (Jenkins et al., 2021; Molinari et al., 2021). Technological solutions originating from SDN2 may or may not be ruled as GMO under the legislation of most countries in the Americas, with the analysis made on a case-by-case basis. In addition, the major parameter to classify SDN2-based products as GMOs is the presence of exogenous DNA in the final product. In the case of SDN3 systems, due to the complexity of the genetic elements introduced in the recipient genome, its derived products are frequently qualified as GMOs, although being assessed case-by-case, as well as considering the origin of the DNA used (Molinari et al., 2021).

A different position was adopted by some countries of the European Union and New Zealand. So far, they decide that plants obtained through gene editing will follow the same criteria applied to GMOs, regardless of the genome editing strategy employed (Jenkins et al., 2021). The People’s Republic of China, despite its outstanding role in the world trade of commodities, has not yet launched regulatory policies for the evaluation of edited plants. These singularities in terms of legislation between countries seem to be linked with different economic aspects, social practices and behaviors, and also political backgrounds. Nevertheless, non-compatible regulatory processes are problematic for international trades, especially in the case of agricultural commodities (Entine et al., 2021). The scientific community, in general, has argued and supported a global level alignment of regulatory policies, which should preserve and strengthen general biosafety requirements, while converging towards the exclusion of some edited bioproducts from the scope of GMOs, depending on the genome editing strategy used. The main point is that whether the obstacles imposed for risk assessment of the edited products were larger than the risks, it surely will discourage innovation, due to increments of costs and time for the technology commercial release. Moreover, in countries where legislation considers that certain products of gene editing may be excluded from GMOs’ scope, there has been a remarkable growth in the number of startups and small and medium-sized biotechnology companies (Entine et al., 2021). Ultimately, it would benefit farmers and final consumers with a wide range of technologies generating superior agronomic traits and better nutritional quality agricultural products.

On the other hand, for the use of topical RNAi-based products in agriculture, worldwide regulatory aspects are still in infancy. In many countries, genetic engineering approaches based on this new technology do not fall within the legislation scope applied to GMOs, nor in the legislation applied to conventional chemical and biological pesticides. Due to its potential advantages, manifold studies on plant protection have been carried out aiming at the development of topically-applied RNAi-based bioproducts.

In 2019, the scientific, industrial and governmental communities gathered at the conference of the Organization for Economic Cooperation and Development (OECD, Paris, France), to discuss various aspects of the technology, and guidelines for risk assessment on human, animal, and environmental health were settled (Mendelsohn et al., 2020). One of the most prominent considerations that emerged from this conference was the strong recommendation to carefully analyze the potential off-targets in the risk assessment of these technologies. The availability of *in silico* tools and the growing genomic data annotation for several species have enabled researchers to identify efficient and specific small RNA molecules, including dsRNAs, reducing the risks of off-targets. This is a clear advantage of that technology over the routinely applied chemical pesticides with a broad spectrum of action, hence, affecting also non-target species (Taning et al., 2020). Moreover, it is recommended that risk analysis likewise look over the lifecycle of RNAi-based products in varied environmental conditions, which in many situations may require reapplication (Mendelsohn et al., 2020).

Pre-existing regulatory frameworks for chemical pesticides and bio-inputs risk assessment in different countries could be used as a basis for evaluating products from RNAi, as long as the specificities of this technology are respected. It is worth noting that if the design and development of these products are performed carefully and rigorously, these technologies might revolutionize with an effective and safe basis to manage pests, weeds, and pathogens effectively.

## 5 Case Studies and Prospects on the Horizon

To date, no topical RNAi-based herbicide/pesticide has been used commercially. However, numerous patents involving topical RNAi for use in agriculture have been applied (Mat Jalaluddin et al., 2019), showing the importance of a worldwide definition of the regulation of these technologies.

Actually, there are commercially approved RNAi-based transgenic crops, like the RNAi insecticidal maize, the soybean with improved fatty acid profile, the non-browning Arctic^∗^ apple, and the low lignin alfalfa (Mat Jalaluddin et al., 2019). However, the whole process of the development and the commercial approval of genetic engineering plants is slow, costly, and for various species very difficult to achieve. Besides, transgenic plants face various regulatory barriers since the first genetically engineered plant was approved in 1994 (Smyth, 2020). It is expected to achieve endogenous plant gene silence using dsRNA at a low cost when compared to GMOs development (Das and Sherif, 2020). In addition, RNAi-based technology with topically applied dsRNA presents low toxicity, and it is species-specific and designed to minimize off-target impacts. Only closely related species to the target presents more risk to be susceptible due to genetic similarity, whereas risks to human health and the environment are very unlikely (Fletcher et al., 2020).

Advances in exploring the use of RNAi technology for crop protection are enabling research results to be transformed into products that are reaching the market. After the commercial release of the first plant expressing dsRNA for pest control (Smart-Stax PRO “MON87411”), Rodrigues et al. (Rodrigues et al., 2021) announced the application for registration of the first sprayable biopesticide based on dsRNA (Ledprona^∗^) intended for the control of the Colorado potato beetle (*L. decemlineata*). The RNAi-based biopesticide is in the process of being registered by the United States Environmental Protection Agency, and *in vivo* tests showed that the Ledprona has an efficiency similar to the Spinosad^∗^ insecticide. In Brazil, the Evolluta Agro Biotecnologia Ltda. intends to launch the product “EVO-201A,” based on the dsRNAs topical application to control *S. frugiperda* and *Helicoverpa armigera*. The product has already been classified by the Comissão Técnica Nacional de Biossegurança (CTNBio) as a non-GMO (CTNBio, 2020). As it is a product under development, no data were revealed regarding the method of dsRNA delivery or the efficiency in control, but it represents a great advance for the development of products based on the dsRNA topical application in agriculture. These events have reinforced the discussion about the environmental safety of the technology for this application.

On the other hand, a large number of plants with an edited genome by CRISPR/Cas are released for cultivation all around the world, in particular United States and Canada. Nowadays, 37 genetically edited organisms using CRISPR/Cas technology have been cleared (i.e., designated as non-regulated product) by the U.S. Department of Agriculture’s Animal and Plant Health Inspection Service (USDA APHIS), being the vast majority composed of plants (USDA APHIS, 2022). In 2016, The Paris mushroom (*Agaricus bisporus*) was the first organism edited using the technology CRISPR/Cas to be designated by the USDA as non-regulated. This product, which normally displayed the darkening of tissues, after the knockout of the polyphenol oxidase (PPO) gene, showed a reduction in the darkening of tissues by 30% and an increase in its shelf life of mushroom (Waltz, 2016). Days after the edited mushroom being cleared by the USDA, the “waxy corn” cultivar, with starch composed entirely of amylopectin, received the same designation as non-regulated product by USDA (Gao et al., 2019; USDA APHIS, 2022). Waxy corn is extremely important for the food, paper, and adhesives industry in the United States, where 2.1 million tons/year are produced in an area of 202.3 thousand hectares (Gao et al., 2019). These and other edited plants and products have been released to commercialization after being exempt of regulation, such as a soybean with high oil and protein content; corn edited to increase drought tolerance and yield stability; plants edited for fungal, bacteria, and herbicides resistance as well as a plant with adapted architectures to different cropping systems (Turnbull et al., 2021; USDA APHIS, 2022). It was in Japan that, for the first time, a product with a genome edited by CRISPR/Cas was released for direct consumption—the tomato variety “Sicilian Rouge High GABA.” This variety has been available in Japanese supermarkets since September 2021 (Newscientist, 2021). Tomato plants naturally contain high levels of gamma-aminobutyric acid (GABA), a beneficial amino acid used for the treatment and prevention of chronic disease that affects the population. The knockout of an auto-inhibitory domain that regulates enzymatic glutamate decarboxylase (GAD) using CRISPR-Cas technology, specifically the knockout of SlGAD2 and SlGAD3 genes, resulting in the occurrence of plants with a 5- to 7-fold greater capacity to produce GABA (Nonaka et al., 2017). The “Sicilian Rouge High GABA” tomato variety is one of the few products already available for consumption and represents an easy and realistic way for consumers to improve their daily diet.

In Brazil, the first plant edited by CRISPR/Cas released for cultivation was the waxy corn from Corteva in 2018. Furthermore, Brazilian researchers developed (through DNA-transgene free CRISPR genome editing) the first non-GMO sugarcane in the world to be considered as non-GMO on 10th December 2021, according to the CTNBio—Normative Resolution 16 (RN 16) (CTNBio, 2018). The sugarcane varieties (Flex and Flex II) offer higher cell wall digestibility and higher sucrose content in plant tissues, respectively. More recently, the soybean edited with low raffinose was also considered as non-GMO by CTNBio on 9th March 2022. Also in South America, a non-GMO potato with reduced enzymatic browning was obtained by the knock-out of a tuber specific polyphenol oxidase (González et al., 2021) (Res. NO-2020-65450768-APN-SABYDR#MAGYP). This variety is under field trials for cultivar registration as a conventional breeding product in Argentina.

Finally, the long-lasting coupling between scientific research and biotechnology has been leading to unprecedented improvements in agricultural products, represented in this review mainly by plant crops, commodities responsible for feeding the globe. CRISPR/Cas- and RNAi-based technologies have revolutionized science and biotechnology due to their high precision, versatility, and relative ease of use, with factual agricultural bioproducts already on the market shelves. Beyond gains in productivity and profitability, these new cultivars (adapted to a broader range of adverse conditions, resistant to diseases and herbicides), eco-friendly bio-pesticides, and all derivative biotechnological solutions hold great potential to solve critical agriculture and environmental issues worldwide, likewise ensuring a sustainable global food supply. However, as reasoned throughout the manuscript, there are still crucial challenges (e.g., delivery, uptake, and stability of the components) and relevant safety issues (e.g., off-/non-target effects and toxicity) to be addressed for a full bench-to-field biotechnological transition. Fortunately, the remarkable fast-paced expansion of both technologies generates an ambience of permanent improvement, which positively impacts the developmental progress of these next-generation crop protection bioproducts. Furthermore, manifold research groups highlight the key role of nanotechnology in the creation of transformational tools suitable to overcome most of the above-mentioned challenges, faced by CRISPR- and topical RNAi-based solutions. Although fine-tune adjustments are still required to overcome inherent technical bottlenecks, it seems that the greatest challenge to be faced towards the full usage of those technologies in modern agriculture is linked with social and political matters (Sprink et al., 2016). Nevertheless, the scientific community, through its inherent transparency and commitment, play key role in the desired convergence of global regulatory landscapes, also in supporting public perception and trust, translating into positive impacts in regulatory policy approvals related to agricultural bioproducts (Maximiano et al., 2021).
